# Acute stress improves the effectivity of cognitive emotion regulation in men

**DOI:** 10.1038/s41598-020-68137-5

**Published:** 2020-07-14

**Authors:** Katja Langer, Bianca Hagedorn, Lisa-Marie Stock, Tobias Otto, Oliver T. Wolf, Valerie L. Jentsch

**Affiliations:** Department of Cognitive Psychology, Institute of Cognitive Neuroscience, Faculty of Psychology, Ruhr University Bochum, Universitätstraße 150, 44780 Bochum, Germany

**Keywords:** Physiology, Psychology, Endocrinology

## Abstract

Emotion regulation is crucial for coping with stressors but in turn can also be influenced by stress. Initial studies provided mixed evidence showing either beneficial or impairing stress effects on cognitive emotion regulation depending on stress timing, sex or the regulatory strategy. Here, we investigated the impact of acute stress on different emotion regulation strategies in men and women. N = 118 healthy participants were subjected to the Trier Social Stress Test or a control condition after which they completed an emotion regulation paradigm, requiring them to regulate their emotions in response to negative pictures using reappraisal or distraction. Cortisol levels were repeatedly measured to quantify changes in HPA axis activity. Affective ratings and pupil dilation served to measure emotion regulation success and the cognitive effort to regulate emotions. Stress reduced arousal and increased valence and success ratings for reappraisal in men, whereas no significant stress effects were found in women. Moreover, stressed men displayed a significant expansion of pupil diameter during reappraisal suggesting enhanced cognitive regulatory engagement, which ultimately may have led to better emotion regulation outcomes. Cortisol secretion positively correlated with subjective reappraisal success in men, suggesting a glucocorticoid-driven mechanism that may promote emotion regulatory performance in the aftermath of stress.

## Introduction

The capability to cope with emotionally challenging situations by means of cognitive emotion regulation is a central need in everyday life, accounting for psychological functioning and mental health^1,2^. Failures to successfully downregulate negative emotions are mediated through a deficient recruitment of mostly prefrontal emotion regulatory networks^3,4^ and represent a crucial risk factor for the development and maintenance of mental disorders^5^.

 Emotion regulation refers to all automatic and controlled modulations in latency, magnitude and duration of emotional activation quantifiable by experiential, behavioural as well as physiological alterations^6,7^. Imaging data indicates that cognitive emotion regulation relies on a common neural network of control areas involving prefrontal and cingulate cortex regions inhibiting activity in limbic structures^8–10^. Individuals employ different emotion regulation strategies which activate specific brain regions within the core regulatory network^9,11^. Cognitive reappraisal and distraction are commonly considered as being most effective in downregulating negative emotions^7^. Whereas distraction involves directing the attention away from the emotional stimulus, reappraisal aims at reframing the valuation of an emotional stimulus^6^. However, not only do individuals differ in the habitual use of particular cognitive emotion regulation strategies but also in their effectivity to apply them^12^. For instance, a meta-analysis by Webb et al.^7^ identified several moderators of the effectiveness of emotion regulation strategies. Most notably, the presence of emotion induction, the direction and magnitude of emotion regulation effort, the number of emotion regulation attempts and the way emotion regulation is measured as well as interactions with sex appeared to influence emotion regulation outcomes. However, there is still a lack of studies that combine experiential, behavioral and physiological measurements depicting emotion regulation in an integral fashion.

 Stress activates two biological pathways: the sympathetic nervous system (SNS) resulting in a rapid release of catecholamines, such as norepinephrine and epinephrine, and the somewhat slower hypothalamic-pituitary-adrenocortical (HPA) axis leading to the secretion of glucocorticoids (GCs; cortisol in humans^13^). Cortisol acts on prefrontal, cingulate as well as limbic structures^14^ via activations of mineralocorticoid (MRs) as well as glucocorticoid receptors (GRs) in the human brain^15^. Interestingly, these brain regions (prefrontal cortex, amygdala, hippocampus,) are also critically involved in emotion regulatory processes^12,16^. Experimental studies investigating the direct impact of acute stress on emotion regulation processes are scarce. However, there is initial evidence that acute stress impairs the cognitive regulation of conditioned fear responses^17^, presumably mediated by inhibited prefrontal control processes^18^. Importantly, impaired fear regulation in response to stress was positively correlated with alpha-amylase levels (an index of SNS activity^19^) but not with cortisol increase, indicating that the regulatory impairments were primarily mediated via catecholaminergic actions. Supporting this idea, previous research showed that stress indeed caused a decline in prefrontal-based cognitive functions, particularly when testing took place during high SNS activity^20^. Furthermore, there are several studies hinting at a stress-induced reduction of cognitive flexibility in favor of habitual routines^21–23^. Since deliberate emotion regulation could be cognitively demanding, the reduction in prefrontal functioning under acute stress states might thus lead to diminished emotion regulatory success. In line with this hypothesis, first evidence provided by our laboratory^24^ demonstrated that stressed participants relative to controls were less effectively distracted from emotional pictures through a parallel arithmetic task. By contrast, however, stress facilitated the active downregulation of negative emotions via cognitive reappraisal, implying that stress may alter emotion regulation success in a strategy-specific manner. These findings suggest that stress and its associated stress mediators may also exert beneficial effects on the ability to regulate negative emotions. For instance, a recent neuroimaging study from our lab revealed that an oral administration of cortisol indeed facilitated the downregulation of negative emotions via both distraction and cognitive reappraisal by enhancing regulatory activity in prefrontal regions and reducing emotion-related activity in the amygdala^25^. Improvement of cognitive emotion regulation after stress might thus be primarily initialized through glucocorticoids acting on cognitive control and emotion processing regions. This idea corroborates with studies showing that cortisol buffers the increase of negative affect^26,27^ and reduces self-reported fear^28^ in response to a psychosocial stressor. Moreover, a growing body of work has demonstrated that instructed as well as habitual emotion regulation enhances neuroendocrine responses to stress^29–31^. Together, these findings suggest that the somewhat delayed stress-induced increase in cortisol exerts an affect-protective function that is potentially mediated through an enhancement of emotion regulatory capacities in the first place, ultimately helping individuals to cope with upcoming negative emotional states^32^. Contradictory findings regarding the effects of stress on emotion regulation outcomes existing in the literature so far could therefore be explained by the opposing effects of glucocorticoids and noradrenergic activity or the relative dominance of one stress system over the other.

 Sex differences in emotional reactivity, emotion regulation effectivity and strategy choice have been repeatedly reported^24, 33–35^. For instance, McRae et al.^33^ showed that men exhibited less increases in prefrontal activity and greater decreases in the amygdala than women when applying reappraisal to downregulate negative emotions. These findings suggest that men may expend less regulatory effort due to a greater use of automatic emotion regulation leading to enhanced neural efficiency. This idea is in line with evidence showing that men are less emotionally reactive than women^34^. Furthermore, sex also seems to modulate stress-induced alterations in the effectivity to downregulate negative emotions via reappraisal^24^.

 Taken together, the current literature on stress and cognitive emotion regulation provides largely heterogeneous results, suggesting complex interactions between stress, sex, and type of emotion regulation. It therefore still remains unclear, how stress and its associated mediators alters different emotion regulation processes and which factors might be critical in modulating these effects. The present study aimed at filling this gap, investigating whether and how stress and its interactions with sex influence the effectivity of two commonly used emotion regulation strategies—reappraisal and distraction—with a special focus on the time window of glucocorticoid dominance. To this end, we exposed men, women taking oral contraceptives and free-cycling women in the luteal phase to the Trier Social Stress Test or a control condition and subsequently tested them in an emotion regulation paradigm. An increase in cortisol as a marker of HPA axis activity and alpha-amylase as an index of SNS activation served to check successful stress induction. Emotion regulation outcome was assessed via affective ratings. A previous study of our lab demonstrated that pupil diameter is not only influenced by the emotional arousal evoked by negative pictures, but also modulated by the cognitive effort to deliberately downregulate negative emotions^36^. Supporting the latter finding, pupil size increases during the downregulation of negative affect were positively associated with prefrontal activity^37^. Collectively, changes in pupil diameter during cognitive emotion regulation have been shown to mirror both emotion regulation effort and success^36–38^ and was thus included as an additional physiological proxy of emotion regulation processes.

 Recent data favors the idea that stress improves emotion regulation success^24,25^, which might be primarily mediated by cortisol^25,27^. Hence, we hypothesized that stress improves the effectivity to downregulate negative emotions, particularly via cognitive reappraisal, which should be evidenced by reduced subjective arousal, enhanced valence and success ratings as well as altered pupil dilations. In addition, we also expected stress to modulate the ability to downregulate negative emotions via distraction. However, given the lack of research on the effects of stress on cognitive emotion regulation in general and rather mixed findings specifically regarding its impact on distraction, we did not hypothesize a specific direction of this effect. Based on studies showing that cortisol diminishes the subjective experience of negative emotions^25,26^ possibly by strengthened emotion regulation processes, we expected cortisol—but not alpha-amylase—to be positively associated with emotion regulation success. Men typically show a stronger cortisol response to acute stress than women^39^. Consequently, we hypothesized that the impact of stress on emotional downregulation is more pronounced in male participants.

## Results

### Subjective and physiological response to stress

#### Physiological stress response

**Figure 1:**
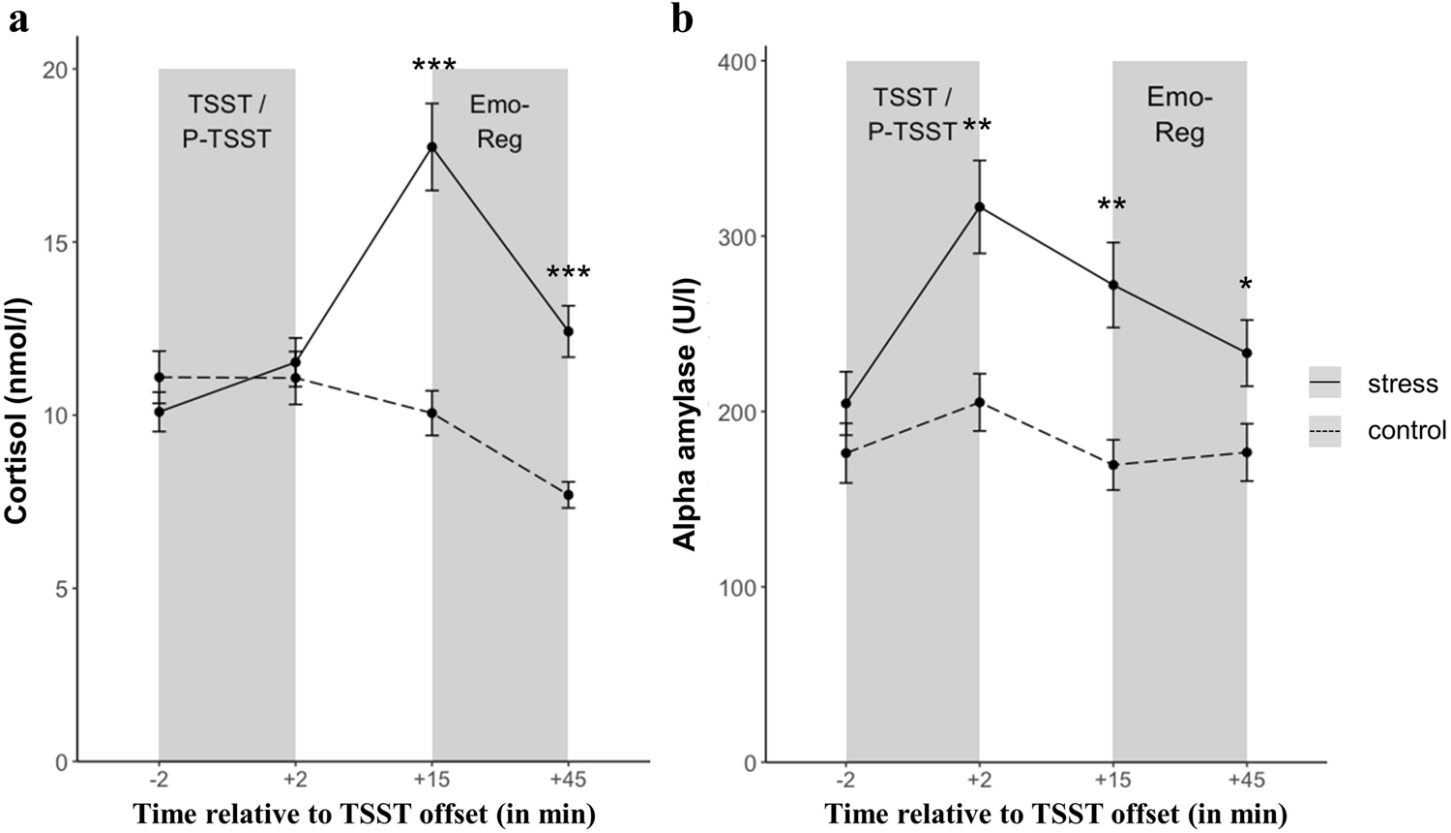
Physiological stress response. Mean (± SEM) salivary cortisol concentrations (**a**) and mean (± SEM) salivary alpha-amylase concentrations (**b**) as a function of stress (stress vs. control). For illustration purposes, raw data is displayed. Time point of the stress manipulation (TSST/P-TSST) and the emotion regulation paradigm (EmoReg) are represented by shaded areas. Significant effects after Bonferroni-corrected post-hoc t-tests are marked as follows: ***p < 0.001; ** *p* < 0.01; * *p* < 0.05.

Figure 1 depicts mean cortisol (panel a) and alpha-amylase responses (panel b) for the stress and control group, showing successful stress induction via the TSST. There was a significant increase in salivary cortisol (main effect of time: *F*(1.59, 166.88) = 29.17, *p* < 0.001; η^2^ = 0.217; main effect of stress: *F*(1, 105) = 12.74, *p* = 0.001; η^2^ = 0.108; stress × time interaction: *F*(1.59, 166.88.01) = 44.14, *p* < 0.001; η^2^ = 0.296) and alpha-amylase concentrations (main effect of time: *F*(2.58, 286.63) = 34.19, *p* < 0.001; η^2^ = 0.235; main effect of stress: *F*(1, 111) = 8.18, *p* = 0.005; η^2^ = 0.069; stress × time interaction: *F*(2.58, 286.63) = 4.40, *p* = 0.007; η^2^ = 0.038) following the TSST as compared to the Placebo-TSST. Post-hoc t-tests showed that there were no differences in salivary cortisol and alpha-amylase levels at baseline (all *p*s > 0.10). However, cortisol and alpha-amylase levels were significantly elevated 15 (both *p*s < 0.002) and 45 min (both *p*s < 0.014) after stress relative to the control manipulation. Significantly higher alpha-amylase levels were also found immediately after stress offset (*p* = 0.002). To ensure that the TSST elicited significant stress responses in all three sex hormone groups, we additionally analyzed differences in delta cortisol and delta alpha-amylase (peak–baseline) between stressed participants and controls for each sex hormone group separately. All three sex hormone groups exhibited significant increases in cortisol concentrations following the TSST as compared to the Placebo-TSST (MALE: *t*(34.26) = − 5.97, *p* < 0.001; FELU: *t*(21.50) = − 2.92, *p* = 0.008; FEOC: *t*(20.32) = -3.86, *p* < 0.001, Table 1). Except for females taking oral contraceptives (*p* = 0.206), males and free-cycling females also showed a significant increase in alpha-amylase levels in response to the stressor (MALE: *t*(23.33) = − 3.09, *p* = 0.005; FELU: *t*(37) = − 2.83, *p* = 0.007, Table 1). Whereas sex hormone groups did not differ in alpha-amylase increase (*p* = 0.259), stressed males exhibited a significantly larger cortisol increase relative to stressed FELU and FEOC (stress x sex hormone interaction: *F*(2, 105) = 7.41, *p* = 0.001; η^2^ = 0.124). No such difference was found in the control groups. Both female groups did not differ in cortisol secretion after stress (*p* = 0.904). Overall, peak cortisol levels were reached 25 min after stress onset (i.e. t _+ 15_), which was immediately followed by the emotion regulation paradigm.

**Table 1:** Mean (± SEM) baseline to peak differences (Δ) in salivary cortisol, alpha-amylase and affective ratings of the stress and the control group for the three different sex hormone groups.

	Δ Cortisol (nmol/l)	Δ Alpha-amylase (U/l)	Δ DAS
	M ± SEM	M ± SEM	M ± SEM
**Stress**			
MALE	13.96 ± 1.87***	115.98 ± 21.29**	0.51 ± 0.13**
FELU	4.55 ± 1.06**	102.85 ± 26.51**	0.60 ± 0.14**
FEOC	4.48 ± 1.07	116.92 ± 26.64	0.35 ± 0.09**
**Control**			
MALE	− 1.61 ± 1.82	22.86 ± 12.29	− 0.12 ± 0.13
FELU	− 0.43 ± 1.34	− 1.91 ± 25.84	− 0.13 ± 0.14
FEOC	− 1.01 ± 1.16	67.84 ± 27.34	− 0.07 ± 0.10

*FELU* free-cycling females,* FEOC* females taking oral contraceptives; significance of pairwise comparisons between stressed MALEs, FELUs as well as FEOCs and the respective controls are marked as follows: ***p < 0.001, **p < 0.01

#### Subjective stress response

Negative affect ratings as assessed by the DAS significantly increased after exposure to the TSST in contrast to the Placebo-TSST (main effect of time: *F*(2, 218) = 8.18, *p* < 0.001; η^2^ = 0.070; main effect of stress: *F*(1, 109) = 13.74, *p* < 0.001; η^2^ = 0.112; stress x time interaction: *F*(2, 218) = 22.45, *p* < 0.001 η^2^ = 0.171). Post-hoc comparisons showed that negative affect was significantly higher in the TSST as compared to the Placebo-TSST group immediately after the stress manipulation (*t*(116) = − 6.56, *p* < 0.001), whereas no group differences occurred at baseline (*p* = 0.100) or 45 min after stress offset (*p* = 0.315). All three sex hormone groups showed a significant increase in negative affect (delta DAS) compared to controls (MALE: *t*(23.57) = − 3.35, *p* = 0.003; FELU: *t*(19.75) = − 3.80, *p* = 0.001; FEOC: *t*(30.45) = − 3.14, *p* = 0.004, Table 1). No differences occurred between the different sex hormone groups (*p* = 0.458).

### Emotion induction and regulation

#### Affective ratings

**Figure 2:**
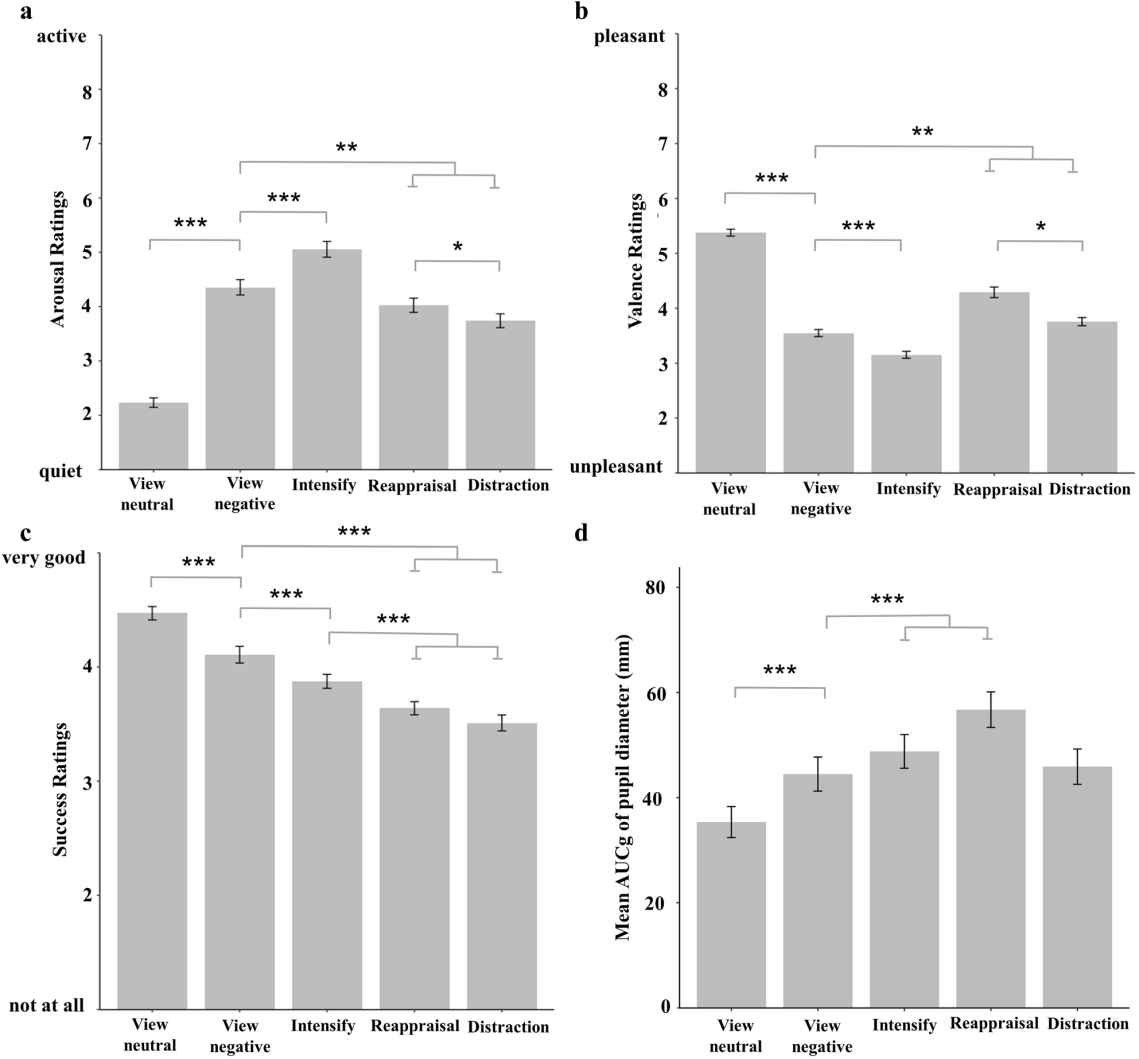
Affective ratings and pupil dilations in the different emotion regulation conditions. Mean (± SEM) subjective arousal (**a**), valence (**b**), and success ratings (**c**), as well as mean (± SEM) pupil diameter (**d**) expressed as the area under the curve with respect to ground (AUCg) for the different emotion regulation conditions. In the view condition, participants rated negative pictures as significantly less pleasant (**b**) and more arousing (**a**) than negative pictures. Moreover, participants showed increased pupil sizes (**d**) after viewing negative than neutral pictures. When downregulating negative emotions via reappraisal or upregulating via intensify, participants exhibited increased pupil dilations (**d**) and rated negative pictures as less arousing (**a**) and more pleasant (**b**) as compared to simply viewing them. Significant effects after Bonferroni-corrected post-hoc t-tests are marked as follows: ***p < 0.001; **p < 0.01; *p < 0.05.

Analyses of affective ratings revealed significant differences in experienced arousal and valence between the emotion regulation conditions (main effect of condition, arousal: *F*(3.48, 389.86) = 197.46, *p* < 0.001; η^2^ = 0.638; Fig. 2a; main effect of condition, valence: *F*(3.29, 367.88) = 213.28, *p* < 0.001; η^2^ = 0.656; Fig. 2b). Post-hoc pairwise comparisons showed that participants rated negative pictures as significantly less pleasant and more arousing than neutral pictures in the *view* condition (both *p*s < 0.001), affirming a modulation of subjective arousal and valence by the emotional content of the pictures. Moreover, both ratings revealed a differential impact of the emotion regulation strategies on subjective emotional responses. When downregulating negative emotions via *distraction* and *reappraisal*, participants rated negative pictures as less arousing and more pleasant as compared to simply viewing them (arousal: both *p*s ≤ 0.001, valence: both *p*s < 0.001). By contrast, when upregulating negative emotions via *intensify*, participants rated negative pictures as more arousing and less pleasant relative to simply viewing them (all *p*s < 0.001). The different emotion regulation conditions also differed regarding the subjectively experienced emotion regulation success (main effect of condition: *F*(3.26, 364.53) = 65.38, *p* < 0.001; η^2^ = 0.369; Fig. 2c), with participants reporting to be significantly more successful in intensifying negative emotions as compared to downregulating them via *distraction* or *reappraisal * (*p* < 0.001).

#### Pupil diameter

Analyses of changes in pupil diameter revealed significant differences in pupil dilation between the conditions (main effect of condition: *F*(2.20, 158.46) = 14.82, *p* < 0.001; η^2^ = 0.171; Fig. 2d). Post-hoc comparisons showed that viewing negative relative to neutral pictures led to a significant increase in pupil dilation (*p* < 0.001), confirming that pupil diameter varies as a function of emotional arousal. When downregulating emotional responses via *reappraisal *or upregulating negative emotions via *intensify,* participants displayed a significantly larger pupil size increase as compared to just viewing them (*p* ≤ 0.024), indicating that the pupil is further modulated by the increase in cognitive effort that is required to up- or downregulate negative emotions.

To check whether control participants have been successful in regulating their emotional responses via the three different strategies, we conducted additional mixed-design ANOVAs including only the control group. These analyses confirmed the result pattern obtained with the whole sample, showing successful induction as well as up- and downregulation of negative emotions via *intensification*, cognitive *reappraisal* and *distraction*. For details regarding the statistical analyses of emotional ratings and pupillary data in the control group, see Supplementary Information A.

### Stress effects on emotion regulation

#### Affective ratings

**Figure 3:**
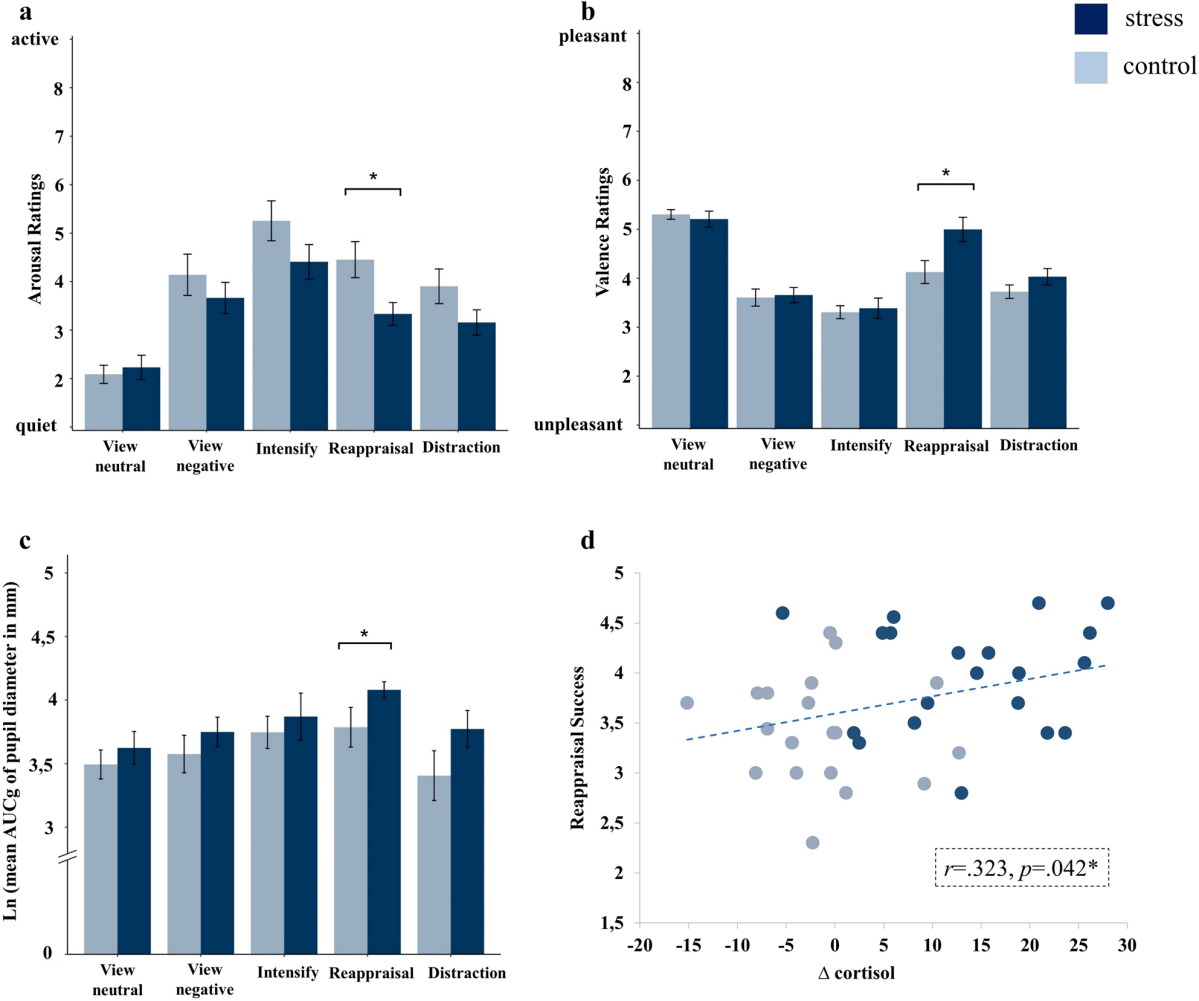
Stress effects on emotion regulation outcome in men. Mean (± SEM) subjective arousal (**a**) and valence ratings (**b**) as well as Ln-transformed mean (± SEM) changes in pupil diameter (**c**) as indexed by the area under the curve with respect to ground (AUCg) of male participants as a function of emotion regulation condition for the stress (TSST) and control (Placebo-TSST) group. Panel (**d**) depicts the relationship between cortisol increase (**∆** cortisol) and success ratings of reappraisal in men. Stressed males exhibited significantly reduced arousal and increased valence ratings as well as pupil dilations after downregulating their emotional response via reappraisal. Delta cortisol values were significantly correlated to success ratings of reappraisal in men. Significant effects after Bonferroni-corrected post-hoc t-tests are marked as follows: *p < 0.05.

A significant stress × sex hormone × condition interaction (*F*(6.96, 389.86) = 2.90, *p* = 0.006; η^2^ = 0.049) indicated that stressed males rated negative pictures as significantly less arousing than controls when applying reappraisal (stress × condition interaction: *F*(3.17, 120.61) = 3.10, *p* = 0.027; η^2^ = 0.075; *t*(38) = 2.54, *p* = 0.015; Fig. 3a). No such stress effect was found in both female groups (stress × condition interaction: both *p*s > 0.648). For valence ratings, the three-way interaction between stress, sex hormone and condition did not reach significance (*F*(6.57, 367.88) = 1.84, *p* = 0.083; η^2^ = 0.032; Fig. 3b). However, exploratory follow-up mixed ANOVAs separately for each sex hormone group revealed a significant stress × condition interaction in males only (*F*(4, 152) = 3.62, *p* = 0.012; η^2^ = 0.087). Post-hoc pairwise t-tests showed that stressed males rated negative pictures as significantly less unpleasant than controls when using reappraisal (*t*(38) = − 2.57, *p* = 0.014). Regarding the emotion regulation success ratings, ANOVA revealed a significant stress x condition interaction (*F*(3.25, 364.53) = 3.87, *p* = 0.008; η^2^ = 0.033). Following up on that, separate post-hoc univariate ANOVAs for the emotion regulation conditions again revealed a significant stress × sex hormone interaction for reappraisal (*F*(2, 112) = 3.92, *p* = 0.023; η^2^ = 0.065), indicating that stressed males were subjectively more successful in reappraising negative pictures as compared to control males (*F*(1, 38) = 10.48, *p* = 0.003; η^2^ = 0.216). No further differences in subjective emotion regulation success occurred between the stress and the control group regarding other emotion regulation strategies (all *p*s ≥ 0.100). To ensure that these effects were indeed driven by stress-induced alterations in emotion regulation and not just confounded by stress-related differences in the view negative condition, we reran the statistical analyses for arousal, valence and success ratings using difference scores between the view negative condition and the four other emotion regulation conditions, respectively (view neutral—view negative, distraction—view negative, reappraisal—view negative, intensify—view negative). Results were highly similar to the original analyses reported above (for details: see Supplementary Information B). In order to check whether random stimulus effects may account for the reported stress effects on emotion regulatory outcomes, we additionally analyzed our data applying a multilevel modelling approach that controls for both random stimulus effects and random participant effects. Linear mixed model analyses revealed similar results like the reported mixed-design ANOVAs (for details: see Supplementary Information D).

#### Pupil diameter

Analysis of the pupillary responses resulted in a significant stress × condition interaction (*F*(2.20, 158.46) = 3.280, *p* = 0.036; η^2^ = 0.044). Separate post-hoc univariate ANOVAs for each emotion regulation condition revealed a significant stress × sex hormone interaction for *reappraisal* (*F*(2, 82) = 3.20, *p* = 0.046; η^2^ = 0.072), indicating that stressed males relative to controls displayed larger pupil dilations when downregulating negative emotional responses via *reappraisal* (*F*(1, 26) = 4.63, *p* = 0.041; η^2^ = 0.151; Fig. 3c). No such stress effect was found in females (*p* = 0.141) or regarding other emotion regulation conditions (all *p*s ≥ 0.097). In order to exclude, that the observed effects of stress on emotion regulation might be attributed to potential differences in baseline pupil diameter between the stress and control group, we conducted a t-test with mean baseline pupil diameter as the dependent and stress as the independent variable. Overall, there were no differences in baseline pupil diameter between the stress and the control group (*p* ≥ 0.368).

Additional figures displaying affective ratings and pupil dilation data for stressed FELUs and FEOCs and their respective controls are provided in the Supplementary Information C.

### The relationship between cortisol and emotion regulation success

Correlation analyses showed that the magnitude of cortisol secretion was positively related to subjective emotion regulation success for *reappraisal* in the entire male sample (*r* = 0.323, *p* = 0.042; Fig. 3d). There were no significant correlations between cortisol increase and success ratings for *distraction* or *intensify* (both *p*s ≥ 0.120).

## Discussion

In this study, we investigated the effects of acute stress on the effectivity of cognitive emotion regulation in males, females taking oral contraceptives and free-cycling females. Results revealed that acute stress led to an improvement of reappraisal—but not of distraction—in men, indicated by reduced emotional arousal as well as enhanced valence and success ratings. In line with subjective data, stronger pupil dilations further demonstrated that stressed males were cognitively more engaged during reappraisal, which in turn might have led to better emotion regulatory outcomes. Moreover, cortisol secretion was positively associated with reappraisal success in men, suggesting the stress-induced improvement of cognitive reappraisal to be predominantly driven by a glucocorticoid mechanism.

 The present findings are consistent with previous studies demonstrating that stress and in particular the stress hormone cortisol facilitate emotion regulatory processes, especially via cognitive reappraisal^24,25^. Even though the functional role and precise mechanism of cortisol in emotional processing is still not fully understood, several researchers postulated a cortisol-dependent improvement of negative emotional experience^27^, phobic fear^28^ as well as cognitive emotion regulation skills^25^. For instance, recent imaging data from our lab suggest that stress facilitates the cognitive downregulation of negative emotions via enhanced regulatory activity in prefrontal regions and reduced emotion-related activity in limbic structures such as the amygdala^25^. Consistently, our findings provide further evidence for a positive association between cortisol secretion and emotion regulation success in men. Changes in the activity of the PFC—amygdala pathway seem to be of particular relevance explaining the stress-induced alterations in cognitive emotion regulation^40^. In line with this notion, Henckens et al.^41^ showed that GCs rapidly suppress amygdala responsivity to emotional stimuli. Supporting the latter finding, administration of cortisol acutely reduced anxiety-driven selective attention to threat^42^ emphasizing that GCs may have anxiolytic effects. Interestingly, stronger stress-induced cortisol increases also appear to mitigate the increase in negative affect that is typically observed after stress^26,32^, which might reflect a cortisol-mediated enhancement of emotion regulatory capacities. Together with these findings, our data provide preliminary support for the idea that the beneficial effects of stress on cognitive emotion regulation are mainly driven by glucocorticoid actions promoting prefrontal and inhibiting amygdala activity^25,41^ which in turn may lead to reduced emotional responses.

 In line with subjective data, the present study further revealed that stress increased pupil dilations when applying reappraisal in men. Previous research from our lab^36^ demonstrated that in emotion regulation tasks pupil diameter is modulated by emotional arousal, but also linked to the mental effort required to regulate emotional responses. Therefore, pupil dilation might reflect both emotion regulation effort and success. Our results support this view, showing greater pupil diameter for negative compared to neutral pictures but even a further enhancement of pupil dilation in regulation trials. It has been previously reported, that pupil size increases in response to downregulation of negative emotions were positively associated with prefrontal activity^37^. Stronger pupil dilations during deliberate attempts to cognitively regulate emotions might therefore indicate cognitive engagement in the task, leading to increases in prefrontal activity. In line with this idea, Allaert et al.^43^ could show that stimulation of the left dlPFC led to an increase in pupil dilation during the presentation of negative stimuli, indicating heightened cognitive resource allocation for emotional processing. There are hence several lines of research suggesting that pupil dilation indeed indexes cognitive control^43,44^. Here we could demonstrate for the first time that stress further boosts pupil dilation when cognitively reappraising negative emotions. This pattern together with the improved subjective emotion regulatory outcome suggest that men engage more in reappraising upcoming negative emotions after stress, which in turn might increase the effectivity of downregulating negative emotions with this strategy.

However, there are also studies showing that stress immediately inhibits prefrontal control processes^20^ and impairs the cognitive regulation of fear^17^. Yet, these inconsistencies may be explained by the relative predominance of the two stress pathways and their associated mediators acting on emotion regulatory processes. For instance, it has been shown that heightened functional connectivity between the amygdala and a number of salience network regions after stress was diminished when the β-adrenergic blocker propranolol was administered, whereas inhibition of glucocorticoid synthesis had no effect^46^. Hence, an upregulation of the salience network during high-stress states and the inhibition of prefrontal activity, which is mostly related to stronger emotional experiences^47^, appear to be predominantly driven by stress-induced catecholaminergic actions. Accordingly, acute stress may be linked to impaired cognitive functions particularly when testing takes place within or close to the time window of catecholaminergic actions^18,20,48^. In line with this hypothesis, Raio and colleagues^17^ could show that the stress-induced impairment of cognitive emotion regulation was correlated with heightened alpha-amylase levels (a marker of sympathetic activity) but not with cortisol. In contrast to the somewhat slower HPA pathway, SNS effects are initialized almost instantly after stress onset^13^. Given that participants underwent the emotion regulation paradigm 30 min after stress onset in the present study, the beneficial effects of stress on cognitive reappraisal might be mainly driven by glucocorticoid actions that come into play after some delay. Consistently, we found cortisol secretion to be positively associated with the success of downregulating negative emotions via cognitive reappraisal. GCs have been shown to actively contribute to the downregulation of salience network activity and an upregulation of the executive control network^20^. Moreover, they are critically involved in restoring homeostasis and normalizing brain activity following stress^15^. It is therefore reasonable that catecholamines may rapidly impair cognitive emotion regulation during acute stress states, while cortisol helps to restore prefrontal functions thereby also promoting emotion regulatory processes as soon as acute stress subsides. For future studies it will be thus of utmost importance to examine the different contributions of the SNS and HPA axis to emotion regulation outcomes.

To the best of our knowledge, the study reported here, is the first experiment to investigate differences in the influence of stress on emotion regulation between males, free-cycling women and women taking oral contraceptives. As hypothesized, we found sex differences in cortisol reactivity to psychosocial stress and its influence on emotion regulation performance. In line with previous studies^39,49^, men exhibited a stronger stress-induced cortisol secretion compared to women. Given the well-established inverted U-shaped dose–response curve between GCs and learning or memory^50,51^, it can be speculated that beneficial effects of stress on reappraisal rely on a certain magnitude of cortisol release. Supporting the idea of an U-shaped dose–response function between GCs and emotion regulation outcomes^25^, we found a positive correlation between cortisol secretion and success of reappraisal in men. Hence, our findings may be suggestive of a dose-dependent glucocorticoid-mediated improvement of cognitive reappraisal, which appear to be attributed to a stronger cognitive engagement to use this strategy in the aftermath of stress. Future work using different dosages of hydrocortisone to investigate its influence on emotion regulation outcomes is clearly needed.

Of note, a stress-induced increase in the effectivity of cognitive reappraisal was found only in men, while stress did not affect emotion regulation in both female groups. Previous work already reported sex-dependent stress effects on emotion regulation processes, in particular on cognitive reappraisal^24^. Furthermore, cortisol diminished subjective emotional responses towards negative pictures in men but not in women^25^ supporting the idea that men may profit from the beneficial effects of stress and glucocorticoids on emotion regulatory processes to a greater extent than women. This result pattern is in line with a growing body of literature showing that stress effects on emotion and cognition are more pronounced in men than in women^52–56^. Stress upregulates sex hormones^57^ which have been shown to alter the physiological stress response and in turn may also affect cognitive functions through direct and indirect effects on GRs^58^. Sex-specific effects of stress on cognitive functioning are most likely driven by interactions between cortisol and sex hormones, such as estrogens, gestagens and androgens^56,59,60^. For instance, estradiol has been shown to decrease GR expression and to inhibit its functionality^61^, potentially explaining sex differences in glucocorticoid responsiveness and stress reactivity. In addition, elevated progesterone levels have been associated with a decreased sensitivity of women to stress effects on cognitive functions^60^. In this study, women displayed weaker cortisol responses to stress than men and the stress-induced improvement of cognitive reappraisal was restricted to men, who typically exhibit lower estradiol and progesterone levels than females. It might be therefore reasonable that the missing stress effect in women was driven by complex interactions between sex-specific hormones and glucocorticoids primarily acting on GRs in prefrontal and limbic regions. Furthermore, under rest, males have been shown to exhibit less pronounced increases in prefrontal regions and greater decreases in the amygdala relative to females when applying reappraisal^33^. Therefore, males may usually expend less effort to downregulate negative emotions via this strategy. In this study, stress prompted an increase in cognitive engagement to reappraise negative stimuli in males, possibly compensating the inhibition of prefrontal activity and executive functions under stress^18^. Future imaging studies in combination with eye tracking are warranted to investigate sex-specific effects of stress on cognitive engagement and prefrontal activity while downregulating negative emotions.

Besides a number of strengths, such as the comparison of three different hormonal status groups and the inclusion of pupil dilation as a physiological measurement of emotion regulation processes in addition to subjective ratings, some limitations have to be noted. First, not only does stress initiate the secretion of cortisol, but also of monoamines and neuropeptides each having its own spatial and temporal domains of release and action^13^. In view of complex interactions between these stress mediators, the present study cannot provide direct evidence about the underlying mechanisms of stress effects on cognitive emotion regulation. Future pharmacological studies are needed to specify the causal link between cortisol and changes in cognitive emotion regulation outcomes. Second, pupil diameter has been shown to represent both emotional arousal and the cognitive effort to control emotional responses^36,43^. Since pupil dilation is not valence-specific^62^, pupil size increases may therefore either be interpreted as an increase in emotional arousal or emotion regulatory engagement. To reduce this ambiguity, future studies would benefit from including complementary physiological measurements, such as the startle reflex (an index of valence^63^), skin conductance response (an index of arousal^64^) or changes in heart rate variability (an index of emotion regulation success^65^).

In conclusion, our findings demonstrate that acute stress improves the downregulation of negative emotions via cognitive reappraisal in men. Pupillary measures further indicated that this stress-induced improvement of reappraisal in men might be a result of increased cognitive engagement to use this strategy after stressful episodes. Reappraisal success was positively linked to the increase in cortisol in men, indicating that the beneficial effect of stress on cognitive reappraisal might be predominantly mediated by a glucocorticoid mechanism. The sex-specific stress effects on subjective affective ratings and pupil dilation offer interesting insights into the interaction between stress, sex and emotion regulation, contributing to an enhanced understanding of sex differences in vulnerabilities to stress- and emotion-related mental disorders.

## Materials and methods

### Participants and design

A total of 118 participants (40 males and 78 females) ranging in age from 18 to 37 (*M* = 24.25, *SD* = 4.29) with a normal Body Mass Index (BMI) ranging between 18.0 and 27.8 kg/m^2^ (*M* = 22.12; *SD* = 2.38) were recruited via advertisements in social media networks, mailing lists and notice boards throughout the Ruhr University Bochum and surroundings. Exclusion criteria checked beforehand in a standardized telephone interview comprised chronic and acute illnesses, history or current medical or psychological treatment, drug use including smoking, as well as previous experiences with the current stress protocol or emotion regulation paradigm. All participants had normal or corrected-to-normal vision (max. ± 1.5 diopters). Due to well-established effects of sex hormones on stress hormone release^39^ as well as on emotional reactivity^34^ and emotion regulatory processes^33^ we included men, free-cycling women in their luteal phase as well as women taking oral contraceptives (OC). Cycle phase was assessed via self-report and luteal phase defined as nine to three days prior to the next menses^55^. OC women were required to have been taken OC (only monophasic preparations with an ethinylestradiol and a gestagenic component) for at least 3 month and were tested during the active pill phase to reduce potential influences of circulating sex hormones across the menstrual cycle^52^. Participants were randomly assigned to the stress and control condition, which did not differ in age (*p* = 0.651), BMI (*p* = 0.765), psychopathological symptoms (*p* = 0.902), and habitual use of reappraisal (*p* = 0.676) or distraction (*p* = 0.438) as assessed with the emotion regulation inventory (ERI). The study was conducted in accordance with the Declaration of Helsinki and all participants provided written informed consent in accordance with procedures approved by the local ethics committee at the Ruhr University Bochum and were reimbursed with 20 Euro.

### Experimental procedure

**Figure 4:**
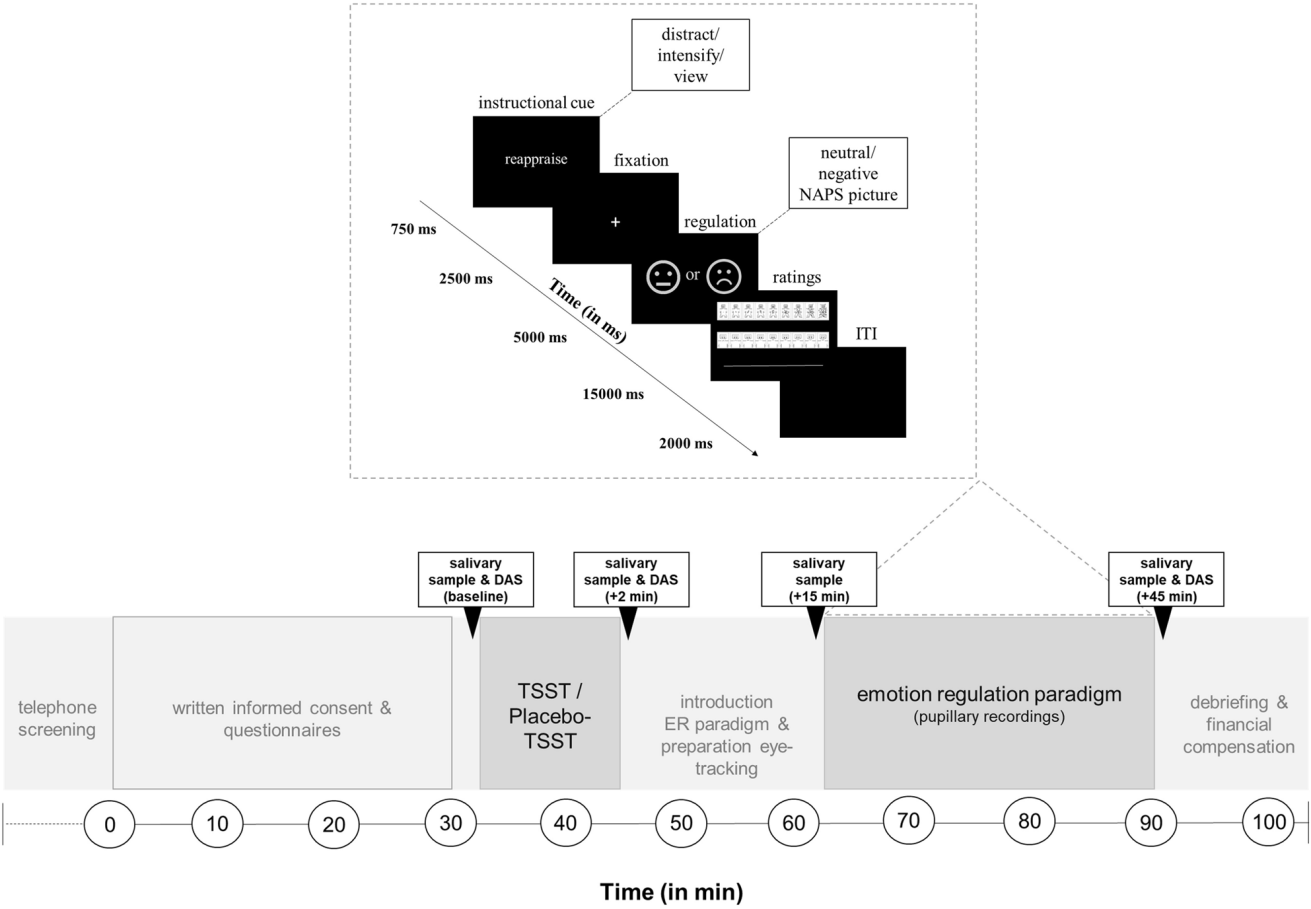
Experimental procedure and emotion regulation paradigm. Participants provided four saliva samples and three affective ratings throughout the experiment (sampling time points for saliva and DAS are highlighted by dashed boxes: baseline, + 2, + 15, + 45 min and baseline, + 2, + 45 min after TSST/Placebo-TSST offset, respectively). In the emotion regulation paradigm, participants were asked to regulate their upcoming emotions towards negative pictures using one of three different emotion regulation strategies or to simply view negative or neutral pictures, which were taken from the Nencki Affective Picture System (NAPS^74^).

Participants were asked to refrain from caffeine, nicotine, food and any drinks except for water two hours before the experimental session. Furthermore, we asked them to refrain from sports, drugs and alcohol 24 h prior to the start of the experiment. To control for diurnal rhythm of endogenous cortisol levels^66^, testing took place between 12.30 p.m. and 6.00 p.m. Figure 4 depicts a detailed description of the procedure. After arrival, participants rested for 25 min while reading study information, giving written informed consent and answering questionnaires. After a baseline saliva sample, participants underwent the stress or control condition and were then familiarized with the emotion regulation paradigm and prepared for pupillary recordings. Subsequently, the emotion regulation paradigm started. This interval between stress and emotion regulation was chosen because of the somewhat slower reaction of the HPA axis, which typically leads to peak cortisol levels at ~ 25 min after stress onset^67^. During the emotion regulation paradigm variations in pupil diameter were recorded. Finally, the participants were debriefed and paid their monetary compensation.

### Stress and control manipulation

Participants in the stress group underwent a short version of the Trier Social Stress Test (TSST^68^) which is a reliable method to activate the autonomous nervous system (ANS) as well as the HPA axis. In short, the protocol comprised a 2-min preparation period, a 5-min free speech in front of a reserved panel (one male/one female) and a 3-min mental arithmetic task by counting backwards from 2043 in steps of 17. The Placebo-Trier Social Stress Test^69^ was used as the control condition, which is identical to the TSST in terms of timing and components but without any stress-eliciting factors. In short, participants gave a speech about their last holiday, a book or a movie without being observed and subsequently had to count forward in steps of 15.

To assess the effectiveness of the stress induction, salivary cortisol, alpha-amylase and subjective affect ratings were measured at several time points across the experiment (see Fig. 1). Saliva was collected with Salivette collection devices (Sarstedt AG & Co., Nümbrecht, Germany) and stored at − 20 °C until analysis. Salivary cortisol were analysed on a Synergy2 plate reader (Biotek, Winooski, USA) using commercial enzyme-linked immunosorbent assays (ELISAs; free cortisol in saliva; Demeditec, Kiel, Germany) according to the manufacturer's instructions. Intra- and interassay variability were less than CV 7.77%. A colorimetric test using 2-chloro-4-nitrophenyl-α-maltrotriosoide (CNP-G3) as a substrate reagent was applied to measure salivary alpha-amylase (sAA) concentrations as described elsewhere^70^. Intra- and inter-assay variabilities were below CV 5.28%. The affective stress response was assessed using the Differential Affect Scale (DAS; negative affect factors: sadness, anger, disgust, contempt, fear, shame, guilt; positive affect factors: joy, surprise, interest^59^) on a 5-point likert scale ranging from 1 (not at all) to 5 (very strong) with three time points of measurement (baseline, + 2, + 45 min after stress/control manipulation offset). A summary score for negative affect was calculated as the mean of the associated factor values.

### Emotion regulation paradigm

A slightly modified version of the emotion regulation paradigm^36^ developed by Kanske et al.^9^ was applied. Participants were instructed to regulate their upcoming emotional response towards negative pictures using one of three different emotion regulation strategies or to simply view either negative or neutral pictures. In the reappraisal condition, participants were asked to reduce the intensity of their emotional response by reappraising the displayed situation on the picture to happen either in a pleasant context or with a pleasant ending. Participants were encouraged to vividly imagine all details and consequences of this reinterpreted situation. In the distraction condition, participants were instructed to actively distract themselves from the emotional stimulus by thinking about a neutral situation, which is completely unrelated to the situation presented on the picture. The intensify condition requested participants to increase the emotional response by either putting oneself in the position of the observed person on the picture or imagining all negative consequences of the presented situation. The control condition (view) consisted of attending to a picture (negative or neutral) without manipulating the upcoming emotions. Forty negative pictures were randomly assigned to the three emotion regulation conditions and the view negative condition for each participant individually with each picture presented only once. In line with previous research in this area^25,71–73^, we also included a view neutral condition, consisting of ten neutral pictures as a manipulation check for the successful induction of negative emotions on subjective and physiological levels. Neutral pictures were randomized across all view neutral trials. Trial order was arranged in blocks of five trials per condition with every condition randomly presented once in the first and once in the second half of the paradigm. Each trial started with a 750 ms instructional cue (view, intensify, reappraisal, distraction) followed by a white fixation cross displayed on a grey luminance-matched background for 2,500 ms. Subsequently the picture was presented for 5,000 ms initiating the emotion induction and regulation phase. After each picture presentation, participants rated their emotional response on a 9-point visual analog scale regarding arousal (ranging from 1 = emotionally calm to 9 = emotionally aroused) and valence (ranging from 1 = negative to 9 = positive). In addition, they were required to indicate how successful they were in applying the respective emotion regulation strategy on a 5-point scale at the end of each trial (ranging from 1 = not successful at all to 5 = very good). Every rating scale was displayed for 5,000 ms. An inter-trial interval depicting a black screen was presented for 2,000 ms before the start of the next trial. In order to ensure that participants understood task instructions and got an idea of how to apply the different emotion regulation strategies, the experimenter went through all instructions once again together with the participants and then practiced the different strategies with sample pictures giving corrective feedback if necessary. To further familiarize participants with the trial structure and timing of the paradigm, eight computer-based practice trials (two of each regulation strategy, one view negative and one view neutral trial) were additionally conducted directly prior to the start of the emotion regulation paradigm. Pictures presented during practice were not included in the actual paradigm. Stimulus presentation and behavioral recordings were controlled by MATLAB R2016a (MathWorks Inc. Natick, MA) on an IBM compatible PC running on Windows 7.

All pictures were taken from the Nencki Affective Picture System (NAPS^74^). We created a set of 40 negative pictures (valence: *M* = 3.55, *SD =* 0.71; arousal: *M* = 4.35, *SD* = 1.53) and a set of 10 neutral pictures (valence: *M* = 5.38, *SD* = 0.68; arousal: *M* = 2.23, *SD* = 0.94). Based on normative ratings, negative pictures were significantly more arousing (*t*(47.84) = 25.15, *p* < 0.001) and negative (*t*(48) = − 13.84, *p* < 0.001) than neutral pictures. All pictures were landscape in orientation (1,024 × 768 pixels), matched for content and complexity and displayed in greyscale. Mean luminosity of the selected pictures was matched using the MATLAB R2016a SHINE toolbox (MathWorks Inc.) such that mean luminosity did not vary between the pictures. To control the level of illumination prior to picture onset, a white fixation cross on a grey background (2,500 ms) with the mean luminosity computed across all pictures preceded picture presentation on each trial.

### Pupillometry

Pupillary data were recorded with iView eye-tracking glasses (iViewETG 2.0, SensoMotoric Instruments, Germany) connected to an SMI-ETG recording device (Lenovo X230-Notebook) compatible to the iViewETG software. A high-definition scene camera equipped with an infrared-sensitive eye camera for dark pupil detection measured retinal and corneal reflections obtaining participants` pupil diameter of both eyes. Following a one-point calibration to ensure correct tracking of the pupil, data were continuously recorded at a binocular sampling rate of 30 Hz and a viewing distance of 60 cm from the screen, while participants´ head position was stabilized via a chin rest^61^. To control for divergent light influences, all testing took place in a permanent moderately lit room. Due to technical failure, pupillary data of eight participants (4 males, 4 free-cycling females) could not be analyzed.

#### Analysis of pupillary data

Pupillary data were preprocessed according to routines developed in our laboratory^36^. Recorded data were averaged across both eyes and smoothed with a finite impulse response filter at 6 Hz. Onsets of event-locked segments (instructional cue, fixation cross, picture presentation) were marked for each trial. Trials with pupil size outside a feasible range (i.e. 1.5–9 mm of pupil diameter^62^) were discarded. In order to prevent data loss, outliers in dilation speed were detected individually with a dilation speed filter within a range of 6–30 median absolute deviations (MAD^63^). After having removed dilation speed outliers, we detected gabs resulting from eye blinks, which may cause pupil size underestimation due to eyelid occlusion. We used a MATLAB-based algorithm to discard trials with major eye blinks (> 100 ms) and to correct trials with smaller gabs with linear interpolation. For each participant and each individual trial, baseline pupil size was defined as the average pupil diameter recorded during the 300 ms prior to picture onset, and was subtracted from the pupil dilations during picture presentation. As a measure of total pupillary increase in response to emotional picture presentation we calculated the area under the curve with respect to ground (AUCg) from 2 to 5 s after picture onset^36,37^. For each condition, pupillary data were averaged across 10 trials.

### Statistical analyses

In order to examine the effects of stress on emotion regulation, we used a 2 × 3 between-subjects design with the factors *stress* (stress vs. control) and *sex hormone* [males vs. free-cycling females (FELU) vs. females taking oral contraceptives (FEOC)]. All statistical analyses were performed in IBM SPSS Statistics 20 (Armonk, USA) for Windows with a significance level set to α = 0.05. Dependent variables were checked for normality using Kolmogorov–Smirnov-test, log-transformed when necessary and checked for homogeneity of variance using Levene-test. Greenhouse–Geisser corrected p-values and degrees of freedom were reported when the assumption of sphericity was violated and partial eta square (η^2^) were given as estimations of effect sizes.

Cortisol, alpha-amylase and subjective affect were analyzed using mixed-design analyses of variance (ANOVAs) with the repeated measurement factor time (t_baseline_, t _+ 2_, t _+ 15_, t _+ 45_ for cortisol and sAA; t_baseline_, t _+ 2_, t _+ 45_ for subjective affect). To verify significant stress responses in all three sex hormone groups, we calculated delta cortisol, delta alpha-amylase and delta DAS by subtracting the baseline sample from the peak sample. Next, we analyzed differences in delta values between stressed participants and controls for each sex hormone group by separate t-tests. In order to verify successful emotion induction and regulation and to investigate stress-induced differences in emotion regulation, we conducted mixed-design ANOVAs with the repeated measures factor *condition* (view neutral vs. view negative vs. intensify vs. reappraisal vs. distraction) for affective ratings and pupil diameter. Significant interactions were solved with appropriate Bonferroni-corrected post-hoc tests. Further, we examined the relationship between stress-induced increases in cortisol concentrations and changes in emotion regulation effectivity. Therefore, we correlated delta cortisol with the mean subjective and physiological measures for every emotion regulation condition (view neutral, view negative, intensify, reappraisal, distraction) using Pearson product-moment correlations.

## Supplementary information

Supplementary information

## Data Availability

The data sets analyzed during the current study are available at the Open Science Framework (OSF) under https://osf.io/qwrtx/.

## References

[1] Berking M, Wupperman P (2012). Emotion regulation and mental health: Recent findings, current challenges, and future directions. Curr. Opin. Psychiatry.

[2] Sheppes G, Suri G, Gross JJ (2015). Emotion regulation and psychopathology. Annu. Rev. Clin. Psychol..

[3] Zilverstand A, Parvaz MA, Goldstein RZ (2017). Neuroimaging cognitive reappraisal in clinical populations to define neural targets for enhancing emotion regulation. A systematic review. Neuroimage.

[4] Picó-Pérez M, Radua J, Steward T, Menchón JM, Soriano-Mas C (2017). Emotion regulation in mood and anxiety disorders: A meta-analysis of fMRI cognitive reappraisal studies. Prog. Neuro-Psychopharmacol. Biol. Psychiatry.

[5] Eftekhari A, Zoellner LA, Vigil SA (2009). Patterns of emotion regulation and psychopathology. Anxiety Stress Coping.

[6] Aldao A, Sheppes G, Gross JJ (2015). Emotion regulation flexibility. Cogn. Ther. Res..

[7] Webb TL, Miles E, Sheeran P (2012). Dealing with feeling: A meta-analysis of the effectiveness of strategies derived from the process model of emotion regulation. Psychol. Bull..

[8] Etkin A, Büchel C, Gross JJ (2015). The neural bases of emotion regulation. Nat. Rev. Neurosci..

[9] Kanske P, Heissler J, Schönfelder S, Bongers A, Wessa M (2011). How to regulate emotion? Neural networks for reappraisal and distraction. Cereb. Cortex.

[10] Ochsner KN, Silvers JA, Buhle JT (2012). Functional imaging studies of emotion regulation: A synthetic review and evolving model of the cognitive control of emotion. Ann. N. Y. Acad. Sci..

[11] Kalisch R (2009). The functional neuroanatomy of reappraisal: Time matters. Neurosci. Biobehav. Rev..

[12] Wang M, Saudino KJ (2011). Emotion regulation and stress. J. Adult Dev..

[13] Joëls M, Baram TZ (2009). The neuro-symphony of stress. Nat. Rev. Neurosci..

[14] Dedovic K, Duchesne A, Andrews J, Engert V, Pruessner JC (2009). The brain and the stress axis: The neural correlates of cortisol regulation in response to stress. Neuroimage.

[15] De Kloet ER (2004). Hormones and the stressed brain. Ann. N. Y. Acad. Sci..

[16] Ochsner KN, Gross JJ (2005). The cognitive control of emotion. Trends Cogn. Sci..

[17] Raio CM, Orederu TA, Palazzolo L, Shurick AA, Phelps EA (2013). Cognitive emotion regulation fails the stress test. Proc. Natl. Acad. Sci..

[18] Arnsten AFT (2009). Stress signalling pathways that impair prefrontal cortex structure and function. Nat. Rev. Neurosci..

[19] Nater UM, Rohleder N (2009). Salivary alpha-amylase as a non-invasive biomarker for the sympathetic nervous system: Current state of research. Psychoneuroendocrinology.

[20] Hermans EJ, Henckens MJAG, Joëls M, Fernández G (2014). Dynamic adaptation of large-scale brain networks in response to acute stressors. Trends Neurosci..

[21] Schwabe L, Wolf OT (2013). Stress and multiple memory systems: From ‘thinking’ to ‘doing’. Trends Cogn. Sci..

[22] Fournier M, d’Arripe-Longueville F, Radel R (2017). Effects of psychosocial stress on the goal-directed and habit memory systems during learning and later execution. Psychoneuroendocrinology.

[23] Wirz L, Bogdanov M, Schwabe L (2018). Habits under stress: Mechanistic insights across different types of learning. Curr. Opin. Behav. Sci..

[24] Kinner VL, Het S, Wolf OT (2014). Emotion regulation: Exploring the impact of stress and sex. Front. Behav. Neurosci..

[25] Jentsch VL, Merz CJ, Wolf OT (2019). Restoring emotional stability: Cortisol effects on the neural network of cognitive emotion regulation. Behav. Brain Res..

[26] Het S, Schoofs D, Rohleder N, Wolf OT (2012). Stress-induced cortisol level elevations are associated with reduced negative affect after stress: Indications for a mood-buffering cortisol effect. Psychosom. Med..

[27] Reuter M (2002). Impact of cortisol on emotions under stress and nonstress conditions: A pharmacopsychological approach. Neuropsychobiology.

[28] Soravia LM (2006). Glucocorticoids reduce phobic fear in humans. Proc. Natl. Acad. Sci..

[29] Denson TF, Creswell JD, Terides MD, Blundell K (2014). Cognitive reappraisal increases neuroendocrine reactivity to acute social stress and physical pain. Psychoneuroendocrinology.

[30] Lam S, Dickerson SS, Zoccola PM, Zaldivar F (2009). Emotion regulation and cortisol reactivity to a social-evaluative speech task. Psychoneuroendocrinology.

[31] Roos LG, Levens SM, Bennett JM (2018). Stressful life events, relationship stressors, and cortisol reactivity: The moderating role of suppression. Psychoneuroendocrinology.

[32] Het S, Wolf OT (2007). Mood changes in response to psychosocial stress in healthy young women: Effects of pretreatment with cortisol. Behav. Neurosci..

[33] McRae K, Ochsner KN, Mauss IB, Gabrieli JJD, Gross JJ (2008). Gender differences in emotion regulation: An fMRI study of cognitive reappraisal. Gr. Process. Intergr. Relat..

[34] Bradley MM, Codispoti M, Sabatinelli D, Lang PJ (2001). Emotion and motivation II: Sex differences in picture processing. Emotion.

[35] Goubet, K. E. & Chrysikou, E. G. Emotion regulation flexibility: Gender differences in context sensitivity and repertoire. *Front. Psychol.* **10** (2019). 10.3389/fpsyg.2019.00935 PMC6521736 31143142

[36] Kinner VL (2017). What our eyes tell us about feelings: Tracking pupillary responses during emotion regulation processes. Psychophysiology.

[37] Urry HL (2006). Amygdala and ventromedial prefrontal cortex are inversely coupled during regulation of negative affect and predict the diurnal pattern of cortisol secretion among older adults. J. Neurosci..

[38] van Reekum CM (2007). Gaze fixations predict brain activation during the voluntary regulation of picture-induced negative affect. Neuroimage.

[39] Kirschbaum C, Kudielka BM, Gaab J, Schommer NC, Hellhammer DH (1999). Impact of gender, menstrual cycle phase, and oral contraceptives on the activity of the hypothalamus–pituitary–adrenal axis. Psychosom. Med..

[40] Pagliaccio D (2015). Amygdala functional connectivity, HPA axis genetic variation, and life stress in children and relations to anxiety and emotion regulation. J. Abnorm. Psychol..

[41] Henckens MJAG, Van Wingen GA, Joëls M, Fernández G (2010). Time-dependent effects of corticosteroids on human amygdala processing. J. Neurosci..

[42] Putman P, Hermans EJ, Koppeschaar H, van Schijndel A, van Honk J (2007). A single administration of cortisol acutely reduces preconscious attention for fear in anxious young men. Psychoneuroendocrinology.

[43] Allaert J, Sanchez-Lopez A, De Raedt R, Baeken C, Vanderhasselt MA (2019). Inverse effects of tDCS over the left versus right DLPC on emotional processing: A pupillometry study. PLoS ONE.

[44] Peña-Gómez, C., Vidal-Piñeiro, D., Clemente, I. C., Pascual-Leone, Á. & Bartrés-Faz, D. Down-regulation of negative emotional processing by transcranial direct current stimulation: Effects of personality characteristics. *PLoS One* **6** (2011). 10.1371/journal.pone.0022812 PMC3146508 21829522

[45] Otto AR, Raio CM, Chiang A, Phelps EA, Daw ND (2013). Working-memory capacity protects model-based learning from stress. Proc. Natl. Acad. Sci..

[46] Hermans EJ (2011). Stress-related noradrenergic activity prompts large-scale neural network reconfiguration. Science (80-)..

[47] Oei NYL (2012). Stress shifts brain activation towards ventral ‘affective’ areas during emotional distraction. Soc. Cogn. Affect. Neurosci..

[48] Luethi M, Meier B, Sandi C (2009). Stress effects on working memory, explicit memory, and implicit memory for neutral and emotional stimuli in healthy men. Front. Behav. Neurosci..

[49] Liu JJW (2017). Sex differences in salivary cortisol reactivity to the Trier Social Stress Test (TSST): A meta-analysis. Psychoneuroendocrinology.

[50] De Kloet ER, Oitzl MS, Joëls M (1999). Stress and cognition: Are corticosteroids good or bad guys?. Trends Neurosci..

[51] Joëls M (2006). Corticosteroid effects in the brain: U-shape it. Trends Pharmacol. Sci..

[52] Merz CJ, Wolf OT (2017). Sex differences in stress effects on emotional learning. J. Neurosci. Res..

[53] Andreano JM, Cahill L (2006). Glucocorticoid release and memory consolidation in men and women. Psychol. Sci..

[54] Cornelisse S, van Stegeren AH, Joëls M (2011). Implications of psychosocial stress on memory formation in a typical male versus female student sample. Psychoneuroendocrinology.

[55] Merz CJ (2017). Contribution of stress and sex hormones to memory encoding. Psychoneuroendocrinology.

[56] ter Horst JP, de Kloet ER, Schächinger H, Oitzl MS (2012). Relevance of stress and female sex hormones for emotion and cognition. Cell. Mol. Neurobiol..

[57] Lennartsson AK, Kushnir MM, Bergquist J, Jonsdottir IH (2012). DHEA and DHEA-S response to acute psychosocial stress in healthy men and women. Biol. Psychol..

[58] Shields GS, Sazma MA, Yonelinas AP (2016). The effects of acute stress on core executive functions: A meta-analysis and comparison with cortisol. Neurosci. Biobehav. Rev..

[59] McEwen BS, Nasca C, Gray JD (2016). Stress effects on neuronal structure: Hippocampus, amygdala, and prefrontal cortex. Neuropsychopharmacology.

[60] Schoofs D, Wolf OT (2009). Stress and memory retrieval in women: No strong impairing effect during the luteal phase. Behav. Neurosci..

[61] Krishnan AV, Swami S, Feldman D (2001). Estradiol inhibits glucocorticoid receptor expression and induces glucocorticoid resistance in MCF-7 human breast cancer cells. J. Steroid Biochem. Mol. Biol..

[62] Zaehringer, J., Jennen-Steinmetz, C., Schmahl, C., Ende, G. & Paret, C. Psychophysiological effects of downregulating negative emotions: Insights from a meta-analysis of healthy adults. *Front. Psychol.* **11** (2020). 10.3389/fpsyg.2020.00470 PMC7177019 32372993

[63] Zaehringer J, Falquez R, Schubert AL, Nees F, Barnow S (2018). Neural correlates of reappraisal considering working memory capacity and cognitive flexibility. Brain Imaging Behav..

[64] Matejka M (2013). Talking about emotion: Prosody and skin conductance indicate emotion regulation. Front. Psychol..

[65] Appelhans BM, Luecken LJ (2006). Heart rate variability as an index of regulated emotional responding. Rev. Gen. Psychol..

[66] Guilliams, T. G. & Edwards, L. E. Chronic stress and the HPA Axis: Clinical assessment and therapeutic considerations. *Stand.* **9** (2010).

[67] Dickerson SS, Kemeny ME (2004). Acute stressors and cortisol responses: A theoretical integration and synthesis of laboratory research. Psychol. Bull..

[68] Kirschbaum C, Pirke KM, Hellhammer DH (1993). The ‘Trier social stress test’—A tool for investigating psychobiological stress responses in a laboratory setting. Neuropsychobiology.

[69] Het S, Rohleder N, Schoofs D, Kirschbaum C, Wolf OT (2009). Neuroendocrine and psychometric evaluation of a placebo version of the ‘Trier Social Stress Test’. Psychoneuroendocrinology.

[70] Lorentz K, Gütschow B, Renner F (1999). Evaluation of a direct α-amylase assay using 2-chloro-4-nitrophenyl-α-D-maltotrioside. Clin. Chem. Lab. Med..

[71] Grillon C, Quispe-Escudero D, Mathur A, Ernst M (2015). Mental fatigue impairs emotion regulation. Emotion.

[72] Strauss GP, Ossenfort KL, Whearty KM (2016). Reappraisal and distraction emotion regulation strategies are associated with distinct patterns of visual attention and differing levels of cognitive demand. PLoS ONE.

[73] Feeser M, Prehn K, Kazzer P, Mungee A, Bajbouj M (2014). Transcranial direct current stimulation enhances cognitive control during emotion regulation. Brain Stimul..

[74] Marchewka A, Zurawski Ł, Jednoróg K, Grabowska A (2014). The Nencki Affective Picture System (NAPS): Introduction to a novel, standardized, wide-range, high-quality, realistic picture database. Behav. Res. Methods.

[75] Bardeen JR, Daniel TA (2017). An eye-tracking examination of emotion regulation, attentional bias, and pupillary response to threat stimuli. Cogn. Ther. Res..

[76] Kret, M. E., Tomonaga, M. & Matsuzawa, T. Chimpanzees and humans mimic pupil-size of conspecifics. *PLoS One* **9** (2014). 10.1371/journal.pone.0104886 PMC4139319 25140998

[77] Kret ME, Sjak-Shie EE (2018). Preprocessing pupil size data: Guidelines and code. Behav. Res. Methods.

