# Supplementary material for: Acute stress improves the effectivity of cognitive emotion regulation in men
Source: Sci Rep. 2020 Jul 14;10:11571. doi: 10.1038/s41598-020-68137-5 (PMC7360604; doi:10.1038/s41598-020-68137-5)
Supplement: Supplementary file 1 — Supplementary information [file 41598_2020_68137_MOESM1_ESM.pdf]

Acute stress improves the effectivity of cognitive emotion regulation in men

Katja Langer[1], Bianca Hagedorn[1], Lisa-Marie Stock[1], Tobias Otto[1], Oliver T. Wolf[1], Valerie L. Jentsch[1]*

[1] Department of Cognitive Psychology, Institute of Cognitive Neuroscience, Faculty of Psychology, Ruhr University Bochum; Germany

Supplemental material

Supplementary results

A

Emotion induction and regulation

To check whether control participants were successful in regulating their emotions with the three described strategies (intensify, reappraisal, distraction), we conducted additional mixed-design ANOVAs including the control group only with the repeated measures factor *condition* (view neutral vs. view negative vs. intensify vs. reappraisal vs. distraction) for affective ratings and pupil diameter.

Affective ratings. Analyses of affective ratings revealed significant differences in experienced arousal and valence for the different emotion regulation conditions (main effect of condition, arousal: $F_{(3.22,\ 118.52)} = 91.78$, $p<.001$; $\eta^2 = .621$; main effect of condition, valence: $F_{(3.16,177.18)} = 117.42$, $p<.001$; $\eta^2 = .677$). Post-hoc t-tests showed that control participants rated negative pictures as significantly less pleasant and more arousing than neutral pictures in the *view* condition (both *p*s<.001) indicating successful induction of negative emotions. When upregulating negative emotions via *intensify*, control participants rated negative pictures as more arousing and less pleasant compared to simply viewing them (both *p*s<.001). When downregulating negative emotions via *reappraisal*, control participants rated negative pictures as more pleasant compared to just viewing them ($p<.001$). Applying *distraction*, participants rated negative pictures as significantly less arousing compared to the view condition ($p=.001$). Together, these results confirmed successful up- and downregulation of negative emotions via the three emotion regulation strategies in participants of the control group. The different emotion regulation conditions also differed regarding the subjectively experienced emotion regulation success (main effect of condition: $F_{(3.26,182.38)} = 53.14$, $p<.001$; $\eta^2 = .487$), showing that control participants were subjectively more successful in upregulating negative emotions via *intensify* than downregulating them via cognitive reappraisal or distraction (both *p*s≤.016).

Pupil diameter. Analyses of pupillary data revealed significant differences in pupil dilation between the different emotion regulation conditions (main effect of condition: $F_{(3.75,93.55)} = 10.79$, $p<.001$; $\eta^2 = .241$). Post-hoc pairwise comparisons showed that control participants exhibited significant larger pupil sizes when downregulating negative emotional responses via *reappraisal* as compared to just viewing negative pictures ($p<.001$). These results support the

notion that the pupil dilates as a function of cognitive effort that is required to deliberately regulate negative emotions via reappraisal, a finding reported also in the main manuscript.

B

Stress effects on emotion regulation

To ensure that the stress effects on emotion regulation reported in the main manuscript were indeed driven by stress-induced alterations in emotion regulation and not just confounded by stress-related differences in the view negative condition, we first calculated difference scores between the view negative condition and the four other emotion regulation conditions, respectively (view neutral – view negative, distraction – view negative, reappraisal – view negative and intensify – view negative). We then reran the mixed-design ANOVAs with the repeated measures factor condition (view neutral vs. distraction vs. reappraisal vs. intensify) and the between-subjects factors *stress* (stress vs. control) and *sex hormone* (MALE vs. FELU vs. FEOC) for affective ratings and pupillary data to specifically test for group differences in emotion regulation outcomes.

Affective ratings. A significant stress x sex hormone x condition interaction ($F_{(5.40,302.61)} = 3.58$, $p=.003$; $\eta^2= .060$) indicated that stressed males rated negative pictures as significantly less arousing than controls when applying *reappraisal* (stress x condition interaction: $F_{(2.43,92.40)} = 3.91$, $p=.017$; $\eta^2= .093$; $t_{(38)} = 2.08$, $p=.045$). No such stress effect was found in both female groups (stress x condition interaction: both ps>.572). For valence ratings, the three-way interaction between stress, sex hormone and condition did not reach significance ($F_{(5.38,301.51)} = 1.07$, $p=.068$; $\eta^2= .035$). However, exploratory follow-up mixed ANOVAs separately for each sex hormone group revealed a significant stress x condition interaction in males only ($F_{(3,114)} = 4.07$, $p=.012$; $\eta^2= .097$). Post-hoc pairwise t-tests showed that stressed males rated negative pictures as significantly less unpleasant than controls when using *reappraisal* ($t_{(38)} = -2.70$, $p=.010$). No such stress effect was found in both female groups (stress x condition interaction: both ps>.676). Regarding the emotion regulation success ratings, ANOVA revealed a significant stress x condition interaction ($F_{(2.83,316.93)} = 3.38$, $p=.021$; $\eta^2= .029$). Separate post-hoc t-tests for each emotion regulation condition revealed a significant main effect of stress for reappraisal, indicating that stressed participants were subjectively more successful in reappraising negative pictures as compared to controls ($t_{(116)} = -2.66$, $p=.009$).

C

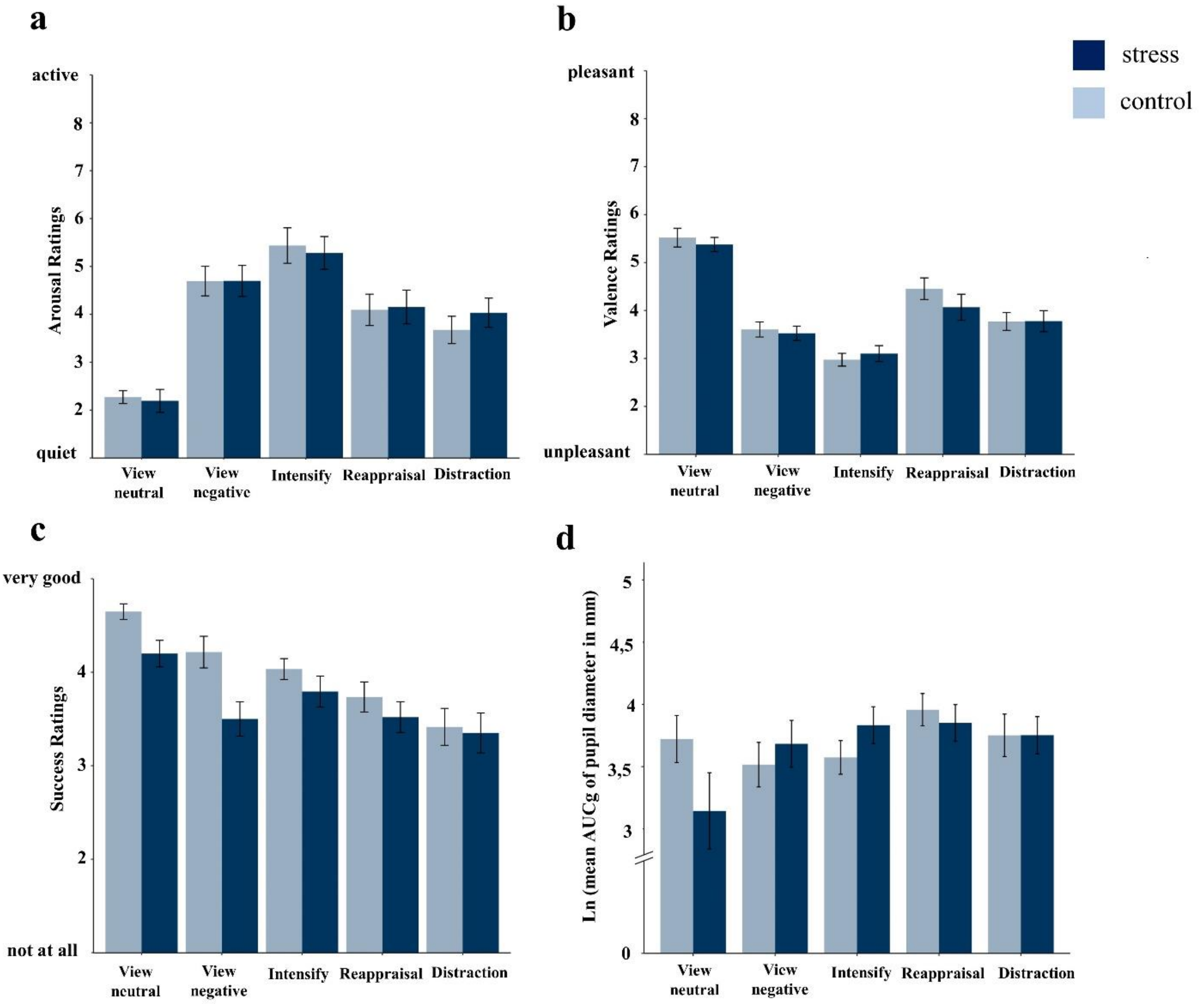

Supplementary Figure S1. Stress effects on emotion regulation outcome in free-cycling females (FELU). Mean (± SEM) subjective arousal (**a**), valence (**b**) and success ratings (**c**) as well as Ln-transformed mean (± SEM) changes in pupil diameter (**d**) as indexed by the area under the curve with respect to ground (AUCg) in free-cycling female participants as a function of emotion regulation condition for the stress (TSST) and control (Placebo-TSST) group. There were no significant differences in emotion regulation outcomes between stressed and control FELUs.

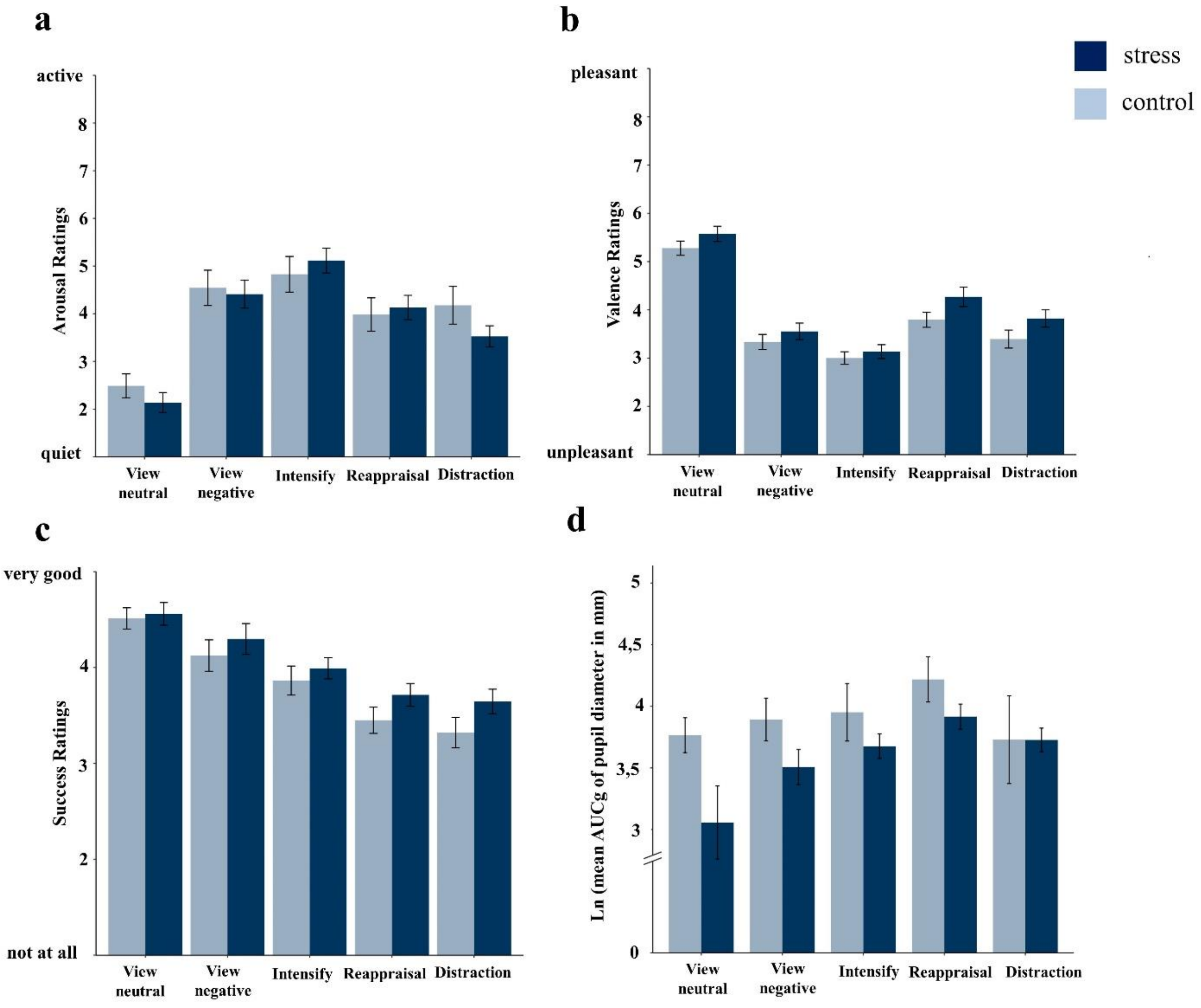

Supplementary Figure S2. Stress effects on emotion regulation outcome in females taking oral contraceptives (FEOC). Mean (± SEM) subjective arousal (**a**), valence (**b**) and success ratings (**c**) as well as Ln-transformed mean (± SEM) changes in pupil diameter (**d**) as indexed by the area under the curve with respect to ground (AUCg) in female participants taking oral contraceptives as a function of emotion regulation condition for the stress (TSST) and control (Placebo-TSST) group. There were no significant differences in emotion regulation outcomes between stressed and control FEOCs.

D

Linear mixed model approach with stimulus and participant as random factors

In order to check whether random stimulus effects may account for the reported stress effects on emotion regulatory outcomes, we reanalyzed our data applying a multilevel modelling approach that controls for both random stimulus effects and random participant effects. Analogous to the mixed-design ANOVAs, we analyzed the effects of emotion regulation condition, stress (0 = control, 1 = stress) as well as sex hormone (0 = male, 1 = free-cycling females (FELU), 2 = female taking oral contraceptives (FEOC)) and their interactions on affective ratings (arousal, valence and success). In order to assume linear changes over the different emotion regulation conditions, we coded the emotion regulation conditions according

to the following expected order (for arousal and valence: 0 = view neutral, 1 = distraction, 2 = reappraisal, 3 = view negative, 4 = intensify; success: 0 = view neutral, 1 = view negative, 2 = intensify, 3 = reappraisal, 4 = distraction). Every linear mixed model (LMM) contained both random factors, the presented stimuli as well as individual differences between the participants, allowing for inter-stimulus and inter-individual differences in affective ratings. Standardized betas (β) are presented as effect size estimates.

The first mixed-model revealed significant differences in experienced arousal, valence and success ratings between the emotion regulation conditions (main effect of condition, arousal: b=.45, β=.21, SE=.02, p<.001, 95% CIs [.410, .486]; main effect of condition, valence: b=-.29, β=-.17, SE=.02, p<.001, 95% CIs [-.330, -.242]; main effect of condition, success: b=-.24, β=-.20, SE=.02, p<.001, 95% CIs [-.283, -.195]) demonstrating the predicted increase in arousal and a decrease in negative valence as well as success over the different emotion regulation conditions. Intraclass correlation coefficients (ICCs) reported in the Supplementary Table S1 provide information about the proportion of variance in affective ratings explained by differences between participants as well as stimuli.

By adding stress and sex hormone consecutively as predictors to the model of arousal ratings, neither the two-level stress x condition interaction nor the three-level interaction between stress, sex hormone and condition reached significance (both ps≥.329). Based on previous studies from our lab showing a sex-dependent influence of stress and cortisol on emotion regulation (Jentsch, Merz, & Wolf, 2019; Kinner et al., 2014) and the reported results in the main manuscript, we conducted exploratory follow-up mixed model analyses separately for each sex hormone group and emotion regulation condition. A significant main effect of stress for *reappraisal* in males only (b=-1.11, β=-.50, SE=.43, p=.014, 95% CIs [-1.986, -.240]; Supplementary Table S2) showed that stressed men rated negative pictures as significantly less arousing than controls when applying *reappraisal*. For valence ratings, both the two-level interaction between stress and condition as well as the stress x sex hormone x condition interaction turned out to be non-significant (both ps≥.190). Exploratory follow-up mixed model analyses separately for each sex hormone group and emotion regulation condition however revealed a significant main effect of stress in males again for *reappraisal* (b=.90, β=.53, SE=.32, p=.008, 95% CIs [.250, 1.559]; Supplementary Table S2). Stressed men rated negative pictures as significantly more pleasant than men in the control group when using *reappraisal*. Analyses of success ratings revealed a significant stress x condition interaction (b=.12, β=.12, SE=.03, p=.001, 95% CIs [.052, .188]), indicating that overall, stressed participants were subjectively more successful in reappraising negative emotions than controls (main effect stress: b=.23, β=.19, SE=.11, p=.043, 95% CIs [.007, .445]; Supplementary Table S2). The three-way interaction between stress, sex hormone and condition however did not reach significance for success ratings (p=.846). In order to explore differences between MALEs, FEOCs and FELUs, we conducted follow-up analyses separately for each sex hormone group. We again found a significant main effect of stress for *reappraisal* only in men (b=.55, β=.46, SE=.16, p=.002, 95% CIs [.215, .878]; Supplementary Table S2), indicating that stress enhanced subjective reappraisal success primarily in males. No stress effects on affective ratings were found regarding the other emotion regulation conditions (all ps≥.109; Supplementary Table S2) or the two female groups (all ps≥.055; Supplementary Table S2). Taken together, LMM analyses therefore supported the results of mixed-design ANOVAs reported in the main manuscript suggesting stress to improve reappraisal success specifically in male participants.

References

Jentsch, V. L., Merz, C. J., & Wolf, O. T. (2019). Restoring emotional stability: Cortisol effects on the neural network of cognitive emotion regulation. *Behavioural Brain Research*, *374*(March), 111880. https://doi.org/10.1016/j.bbr.2019.03.049

Kinner, V. L., Het, S., & Wolf, O. T. (2014). Emotion regulation: exploring the impact of stress and sex. *Frontiers in Behavioral Neuroscience*, *8*(November), 1–8. https://doi.org/10.3389/fnbeh.2014.00397

Supplementary TABLE S1 I Intraclass correlation coefficients (ICCs) of random stimuli and random participants effects with emotion regulation condition as fixed factor.

	**stimuli**	**participants**
**arousal**	.1690	.6208
**valence**	.1915	.1696
**success**	.0150	.2818

Note: ICCs provide information about the proportion (ICC multiplied by 100 = %) of variance in affective ratings explained by differences between participants as well as stimuli.

Supplementary TABLE S2 I Fixed stress effects for affective ratings (arousal, valence, success) with respect to the different emotion regulation conditions for each sex hormone group.

	b	*β*	*SE*	*p*	lower 95 % CI	upper 95 % CI	*b*	*β*	*SE*	*p*	lower 95 % CI	upper 95 % CI	*b*	*β*	*SE*	*p*	lower 95 % CI	upper 95 % CI
**arousal**																		
	**MALE**						**FELU**						**FEOC**					
view neutral	.14	.06	.31	.652	-.48	.76	.02	.00	.27	.945	-.52	.56	-.35	-.16	.32	.282	-.99	.30
view negative	-.46	-.21	.51	.379	-1.50	.58	.03	.02	.43	.939	-.84	.90	-.15	-.07	-.15	.741	-1.07	.77
distraction	-.70	-.31	.43	.109	-1.56	.16	.34	.15	.42	.425	-.51	1.18	-.68	-.31	.43	.120	-1.55	.19
**reappraisal**	**-1.13**	**-.50**	**.43**	**.014**	**-1.99**	**-.24**	-.00	-.00	.46	.999	-.94	.94	.10	.05	.41	.808	-.73	.94
intensify	-.85	-.38	.31	.120	-1.92	.23	-.04	-.02	.48	.933	-1.01	.93	.31	.14	.44	.486	-.58	1.20
**valence**																		
view neutral	-.10	-.06	.19	.591	-.48	.28	-.23	.13	.22	.316	-.67	.22	.30	.18	.21	.159	-.12	.73
view negative	.11	.06	.22	.634	-.34	.56	-.12	-.07	.20	.540	-.53	.28	.27	.16	.22	.221	-.17	.72
distraction	.28	.16	.21	.191	-.14	.69	.03	.02	.03	.924	-.53	.59	.41	.24	.41	.112	-.10	.91
**reappraisal**	**.90**	**.53**	**.32**	**.008**	**.25**	**1.56**	-.42	-.25	.34	.216	-1.10	.26	.51	.30	.26	.055	-.01	1.03
intensify	.08	.05	.24	.734	-.40	.56	.08	.05	.19	.691	-.31	.47	.15	.09	.19	.440	-.24	.53
**success**																		
view neutral	-.09	-.08	.26	.722	-.61	.42	-.47	-.40	.16	.005	-.79	-.15	.05	.04	.16	.747	-.27	.37
view negative	-.05	-.04	.25	.850	-.54	.45	-.78	-.66	.24	.002	-1.27	-.29	.17	.15	.22	.442	-.28	.63
distraction	.29	.24	.25	.183	-.14	.72	-.02	-.02	.28	.947	-.58	.54	.37	.31	.20	.075	-.04	.78
**reappraisal**	**.55**	**.46**	**.16**	**.002**	**.22**	**.88**	-.18	-.15	.21	.413	-.61	.26	.28	.24	.18	.118	-.07	64
intensify	.24	.20	.24	.329	-.25	.73	-.24	-.20	.20	.228	-.63	.16	.12	.10	.18	.492	-.24	.49

Note: Significant main effects of stress on emotion regulation (p<.05) are marked in bold. Stress was coded as follows: 0 = control, 1 = stress. Stress hormone groups: MALEs, free-cycling females in the luteal phase (FELU) and females taking oral contraceptives (FEOC). Arousal ratings ranged from 1 (emotionally calm) to 9 (emotionally aroused), valence ratings ranged from 1 (negative) to 9 (positive), success ratings ranged from 1 (not successful at all) to 5 (very good). Positive regression coefficients (b) indicate an increase in the outcome variable in the stress compared to the control group, whereas negative regression coefficients indicate a decrease.
